# The Use of Fullerenol as a Seed Treatment to Maintain the Intensity of Physiological and Biochemical Processes of Wheat in Control and Low-Temperature Conditions

**DOI:** 10.3390/plants15172704

**Published:** 2026-09-03

**Authors:** Alexander Deryabin, Kseniya Zhukova, Nataliya Naraikina, Ivan Kochetkov, Victoriya Dzhafarova, Yuliya Venzhik

**Affiliations:** K.A. Timiryazev Institute of Plant Physiology, Russian Academy of Sciences, 127276 Moscow, Russia; anderyabin@ifr.moscow (A.D.); zhukova@ifr.moscow (K.Z.); naraikina@ifr.moscow (N.N.); kochetkov@ifr.moscow (I.K.); phytonano@ifr.moscow (V.D.)

**Keywords:** *Triticum aestivum*, fullerenol, seed priming, cell structure, photosynthetic apparatus, gene expression, pro-/antioxidant balance, low temperature

## Abstract

It is of utmost importance to investigate the effect of carbon-based nanostructures on plants in order to fully unlock their potential for enhancing productivity and stress tolerance. In this study, a comprehensive assessment of the effect of pre-sowing treatment of wheat seeds with fullerenol (PHF [C_60_(OH)_24_], 0.1 mg/L) solution on a number of physiological and biochemical parameters in seedlings under optimal and low temperature (LT, 4 °C, 5 days) conditions was carried out. Seedlings primed with PHF differed from control variant in accelerated growth (by 27–34%), increased biomass (by 10%), larger area of chloroplasts and mesophyll cells (by 40%), more intensive photosynthesis (1.5-fold higher), increased content of chlorophyll *a* (by 11%), proteins (by 28%) and ascorbic acid, reduced level of MDA and H_2_O_2_, decreased SOD activity (by 45%) and a higher level of COR-genes (*WCOR15*, *WCOR726*) transcription (*p* < 0.05). Under LT conditions, PHF nanopriming enhanced photosynthesis intensity by increasing chloroplast area, content of chlorophyll *b* (by 22%) and proportion of chlorophyll in LHC (by 11%), as well as increasing the expression of *RBCS* (fourfold higher), content of proline, and COR-gene expression (*p* < 0.05). All these changes are adaptive and expand the adaptive potential of plants. It is concluded that nanopriming with PHF is an active metabolic modulator that reduces ROS generation and enhances cold acclimation of wheat.

## 1. Introduction

In the 21st century, there has been significant progress in the field of nanobiotechnology. Nanomaterials, defined as materials in which at least one dimension is 100 nm or less, play a key role in this advancement. Due to their small size, nanomaterials exhibit an increased surface-to-volume ratio compared with bulk materials, which enhances their biochemical reactivity [1,2,3]. Nanomaterials have significant potential for crop production in unstable climates [4,5]. Carbon-based nanomaterials account for up to 40% of the total quantity of artificial nanoparticles used in agriculture [6]. Among carbon-based nanomaterials, the most common and well-studied is fullerene C_60_. It is a spherical molecule with a diameter of about 0.7 nm. It consists of 60 carbon atoms forming a quasi-spherical geometry [7]. Fullerene consists of 20 hexagonal and 12 pentagonal rings as the basis of an icosahedral symmetry-closed cage structure. Each carbon atom is bonded to three others and is sp^2^-hybridized. However, the limited solubility of fullerenes in water makes it impossible to conduct research on plants and animals [8]. This difficulty was overcome by the addition of hydroxyl groups to the fullerene molecule [9]. Synthesized derivatives of fullerene nanomaterials are called polyhydroxylated fullerenes (PHF) or fullerenols [10,11,12]. Molecules of PHF [C_60_(OH)*_n_*, 2 ≤ *n* ≤ 44] have a diameter of about 1.0 nm and an amphiphilic structure. The hydrophilic shell formed by hydroxyl groups ensures water solubility, while the hydrophobic carbon core provides a high affinity for the lipid components of cellular membranes [13]. As a result, functionalization of PHF with hydroxyl groups has endowed these molecules with good biocompatibility and low toxicity, leading to their broad application in medicine, cosmetology, and biology [9,12]. Numerous studies conducted on animal models have revealed that PHFs exhibit pronounced antibacterial, radioprotective, antioxidant, and cytoprotective activities [10,12,14].

Currently, PHFs are considered to be promising regulators of plants. In low concentrations, they increase crop growth, stress tolerance, and yield [1,15,16]. The study [17] showed for the first time that treating bitter melon (*Momordica charantia*) seeds with a PHF [C_60_(OH)_20_] solution increased biomass yield by up to 54%, fruit length by up to 20%, and fruit quantity by up to 59%. At the same time, the content of two anticancer phytochemicals, cucurbitacin-B and lycopene, was enhanced to 74% and 82%, respectively; the content of two antidiabetic phytomedicines, charantin and insulin, was increased to 20% and 91%, respectively [17]. In another study [18], high concentrations of PHF (50 mg/L and 100 mg/L) administered with a nutrient solution significantly suppressed sunflower growth, relative water content, increased ion leakage, and decreased pigment levels. These doses also slightly impaired photosystem II (PSII) structural and functional integrity and restricted electron transport. At the same time, PHF at a low concentration (10 mg/L) exerted a stimulatory effect on growth and physiological processes of safflower [18]. In *Arabidopsis thaliana*, a solution of PHF [C_60_(OH)_24–26_] acts as a regulator of growth and a modulator of biological processes [19]. Thus, the effect of carbon-based nanomaterials on plants has a hormesis effect—stimulation at relatively low doses and inhibition at higher doses [5].

There are studies in which a key role of various PHFs in enhancing plant tolerance to stressors was demonstrated. For example, a decrease in the level of reactive oxygen species (ROS) and malondialdehyde (MDA) in maize leaves was observed in drought conditions under the influence of PHF [C_60_(OH)_22_·8H_2_O]*_n_* [20]. Priming of *Brassica napus* seeds with PHF [C_60_(OH)_27_] at concentrations of 10 mg/L and 100 mg/L exerted a significant stimulatory effect on seed germination in a 15% polyethylene glycol treatment [21]. Moreover, under water stress conditions, PHF [C_60_(OH)_27_] stimulated plant growth and enhanced physiological and biochemical parameters of the photosynthetic apparatus (PSA) and the antioxidant system in *B. napus*. Foliar application of PHF [C_60_(OH)_24_] contributed to the adaptation of *Beta vulgaris* to drought [22]. In *A. thaliana*, foliar application of PHF [C_60_(OH)_24_] reduced oxidative stress caused by drought [23]. Wheat treatment with PHF [C_60_(OH)_23_⋅12H_2_O] solution at concentrations of 50 mg/L in drought conditions improved seed germination, seedling growth, and antioxidant enzyme activity [24]. In addition, PHF treatment has significantly reduced the negative impact of salt stress in wheat. This was accompanied by an increase in the content of photosynthetic pigments, soluble sugars, the activity of antioxidant enzymes and a decrease in MDA levels [25]. Moreover, foliar spraying with PHF [C_60_(OH)_20_] improved photosynthetic activity and osmolytic accumulation in wheat under salt stress [26]. In cucumber plants, PHF [C_60_(OH)_22–24_] at concentrations of 2 mg/L and 10 mg/L reduced the toxic effects of heavy metals (Cu, Zn), concomitant with an increase in antioxidant activity [27,28].

The method of fullerene delivery to a plant can significantly affect physiological processes. The treatment of plant seeds with fullerenol is known as a unique technology for increasing stress tolerance and plant metabolism [29]. The study showed that pre-sowing treatment of wheat seeds with fullerenol solutions enhances the growth and antioxidant activity of seedlings [30]. Seed treatment with fullerenol had a positive impact on germination, plant growth, and photosynthesis in *Brassica napus* under water stress [21]. Priming with fullerol improved seed germination, seedling growth and the antioxidant enzyme system of wheat under drought [24] and salt stress [25].

The relevance and practical significance of this study are due to the search for new approaches to maintaining crop survival in low temperatures. To date, there are few data on the role of PHF in maintaining basic physiological and biochemical processes in plants at low temperatures. Low temperature is known to be one of the major abiotic factors that affect all aspects of plant life [31]. In this context, we therefore examined, for the first time, the effects of PHF [C_60_(OH)_24_] on plants in optimal and low-temperature conditions. The experimental object was wheat (*Triticum aestivum* L.), a major staple crop worldwide. Wheat seedlings at early developmental stages (in late spring) are often subjected to damaging episodes of recurrent cold, which cause substantial losses in agriculture by reducing both grain yield and quality [32]. Under low-temperature conditions, wheat plant growth slows down, and the main physiological and biochemical processes, the pro-/antioxidant balance, and the gene expression patterns are altered [33,34]. Addressing these challenges necessitates the application of novel approaches, including the use of advanced nanotechnologies.

The purposes of this study were (1) to determine the concentration of the colloidal PHF solution that stimulates the sowing qualities of wheat seeds and (2) to evaluate PHF seed priming-induced changes in growth processes, mesophyll cell structure, parameters of PSA, the pro-/antioxidant balance and the transcript levels of key photosynthesis-associated and COR genes under optimal and low sub-damaged temperature conditions (4 °C, 5 days). The study used seed priming as the most economical and environmentally friendly method of plant treatment.

## 2. Results

### 2.1. Electrokinetic (Zeta) Potential and Particle Diameter of the PHF [C_60_(OH)_24_] Colloidal Solution

**Figure 1:**
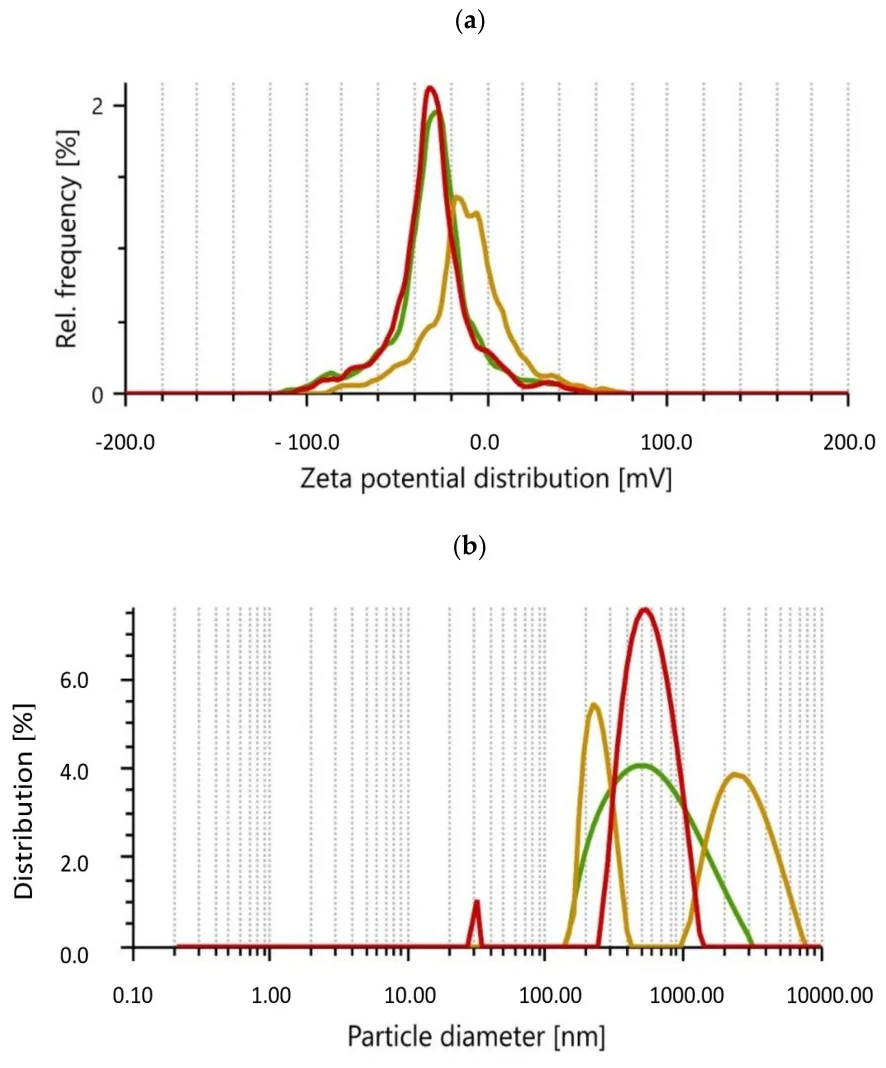
Electrokinetic potential (ζ-potential) (**a**) and particle diameter (**b**) of the aqueous PHF [C_60_(OH)_24_] suspension (0.1 mg/L), measured by dynamic light scattering (n = 3).

The colloidal PHF [C_60_(OH)_24_] solution at a concentration of 0.1 mg/L was characterized using a Litesizer 500 laser analyzer by dynamic light scattering (DLS). The results of ζ-potential measurements and particle diameter for the PHF suspension used in this study are shown in Figure 1. According to the DLS data, the mean ζ-potential value was −32.5 mV, indicating high electrostatic stability of the colloidal system of PHF.

### 2.2. Effect of Seed Priming with PHF on Germination Energy and Germination Rate

**Figure 2:**
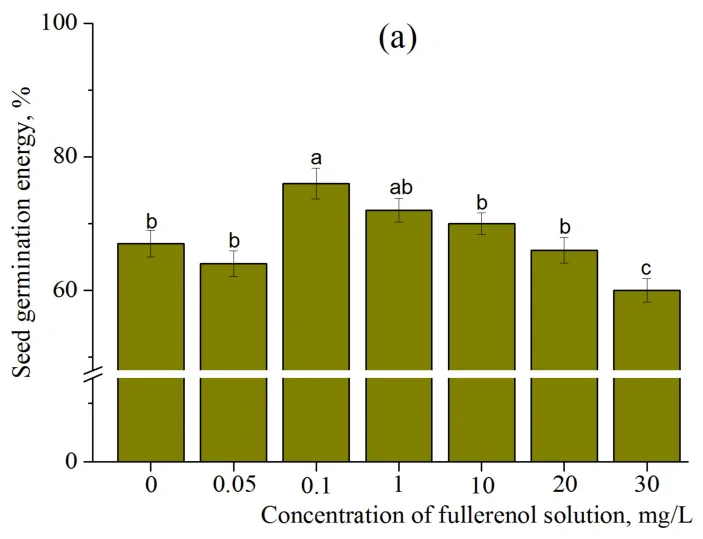

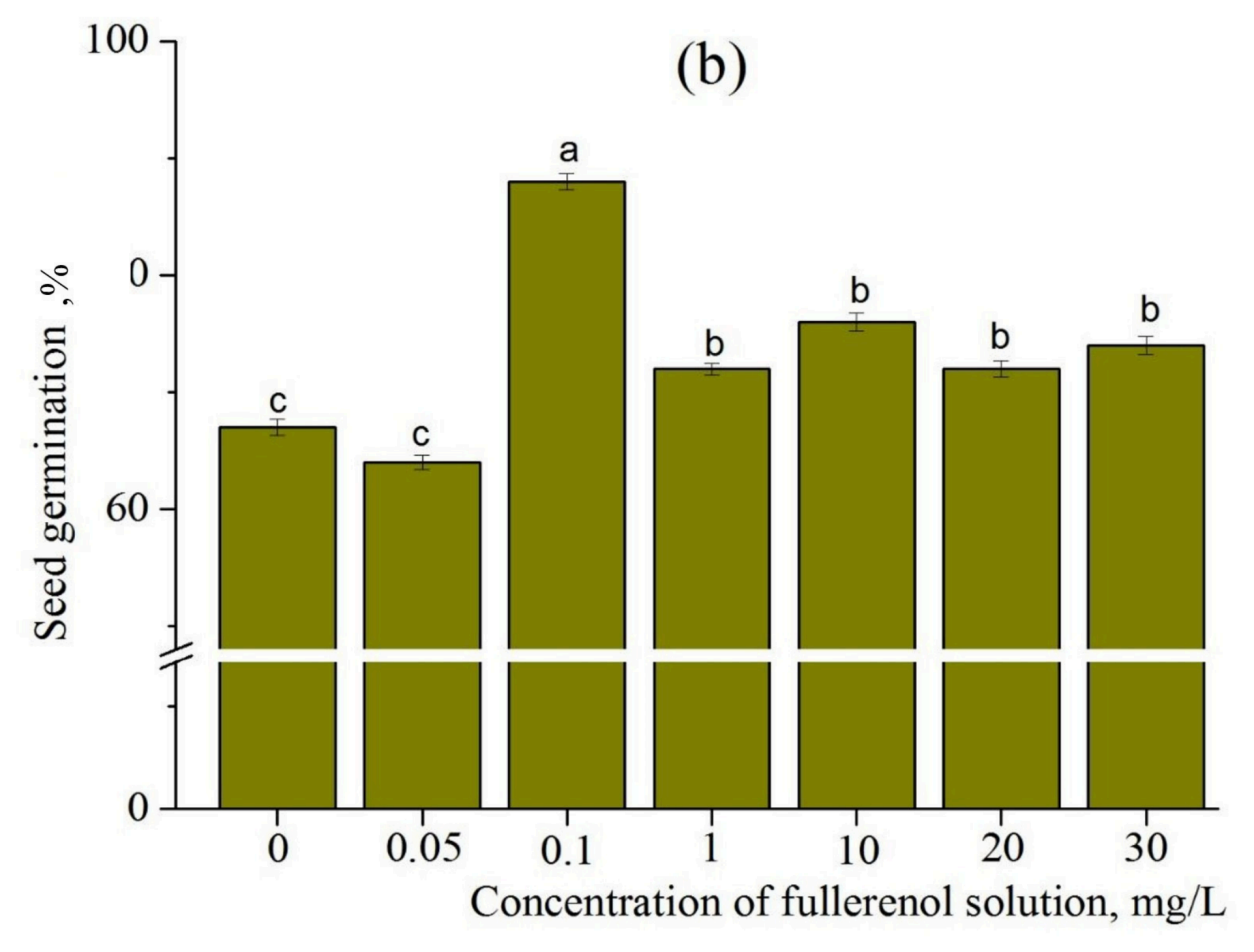
Effect of seed priming with PHF in different concentrations on seed germination energy (**a**) and germination (**b**) of wheat. Mean values ± SE are shown (n = 2). Values that differ significantly at *p* < 0.05 (Tukey’s test) are indicated by different letters.

Concentration tests were used to determine the lowest concentration of PHF that has the maximum stimulating effect on the quality of seed germination (Figure 2). Nanopriming with PHF at a concentration of 0.1 mg/L resulted in the most pronounced stimulating effect on seed germination energy (Figure 2a). In this case, seed germination energy was 9% higher than in the non-primed control. There was a clear trend towards a decrease in seed germination energy with increasing PHF concentration. At the highest PHF concentration (30 mg/L), this parameter was 6% lower than in the control. At the same time, nanopriming with PHF at 0.05 mg/L did not affect seed germination energy.

In seeds primed with PHF at 0.1 mg/L, germination was 21% higher than in the control (Figure 2b). A slight stimulatory (by 5–9%) effect of higher PHF concentrations (1, 10, 20 and 30 mg/L) on seed germination was also noted. Nanopriming with PHF at 0.05 mg/L had no influence on this parameter. Considering the importance of high seed quality indices in agriculture, PHF at 0.1 mg/L—showing the strongest stimulatory effect on both seed germination energy and germination—was selected for subsequent experiments.

### 2.3. Effect of Seed Priming with PHF on Growth Parameters and Seedling Biomass

**Figure 3:**
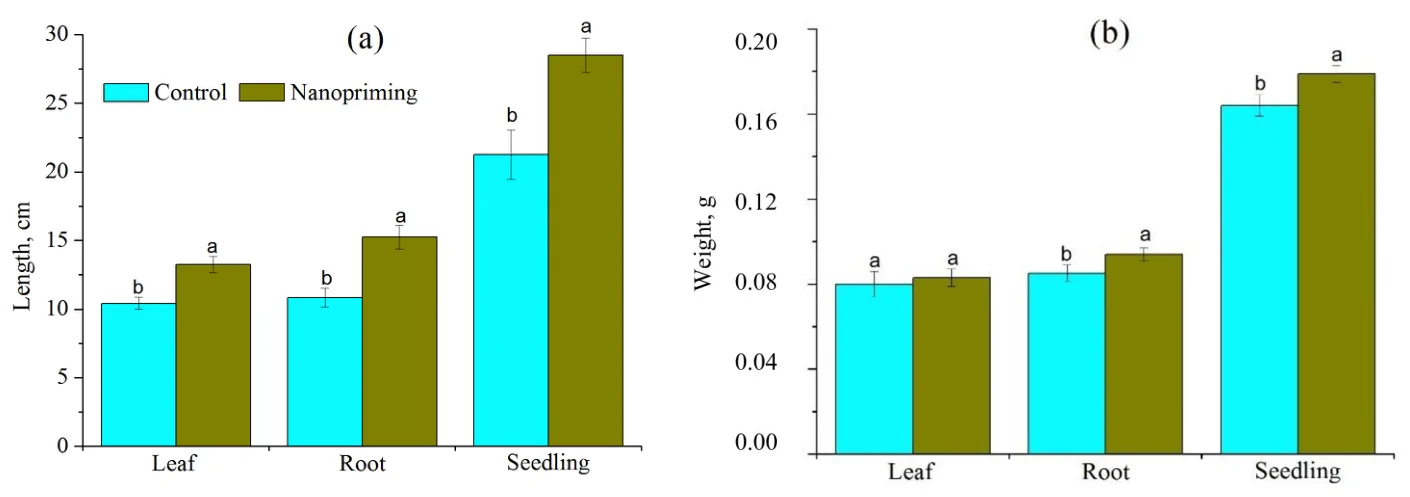
Effects of seed priming with PHF (0.1 mg/L) on morphometric parameters (**a**) and fresh biomass of wheat seedlings (**b**). Mean values ± SE are shown (n = 30). Values that differ significantly at *p* < 0.05 (Tukey’s test) are indicated by different letters.

In Figure 3, the effects of PHF on morphometric traits of wheat seedlings are presented.

The length of leaves, roots, and total length of seedlings treated with PHF was 27%, 40%, and 34% higher, respectively, than in the control. Fresh mass of roots and whole seedlings was increased by 10% in the PHF-primed variant.

### 2.4. Effect of Seed Priming with PHF on Mesophyll Cell Structure of Wheat in Control and Low-Temperature Conditions

Seed priming with PHF affected the structural parameters of mesophyll cells differently under control and low-temperature (LT) conditions (Table 1).

In control conditions, PHF treatment increased cell area due to a 40% increase in cytoplasm volume. At the same time, the chloroplast area was also increased, but the percentage of cell area occupied by chloroplasts within the cytoplasm was decreased to approximately 40%. Under LT, primed plants tended to increase the area of chloroplasts. As a result, the total area of chloroplasts in the cytoplasm was higher in this variant. PHF did not affect the number of chloroplasts per cell either under control conditions or under LT (Table 1).

### 2.5. Effect of Seed Priming with PHF on PSA Activity of Wheat in Control and Low-Temperature Conditions

Nanopriming with PHF affected key physiological and biochemical PSA parameters of wheat seedlings—photosynthetic rate and the quantitative composition of photosynthetic pigments. In PHF-primed seedlings under optimal temperature, the photosynthetic rate was 1.5-fold higher than in the control (Table 2). The content of chlorophyll *a* in primed seedlings was increased by 11%, while chlorophyll *b* and carotenoid content did not differ from the control. Consequently, the chlorophyll *a* to chlorophyll *b* ratio in leaves of PHF-primed seedlings was 30% higher, whereas the level of chlorophyll in LHC was almost 20% lower than in the control (Table 2).

Under LT, the photosynthetic rate in both control and PHF-treated seedlings was decreased, but remained 50% higher in PHF-primed seedlings. Maintaining this level of photosynthesis in primed plants in LT was accompanied by an increase in the content of chlorophyll *b*. In addition, the proportion of chlorophyll in LHC was 11% higher in seedlings treated with PHF under LT.

### 2.6. Effect of Seed Priming with PHF on the Content of Soluble Sugars and Proteins in Wheat Leaves in Control and Low-Temperature Conditions

**Figure 4:**
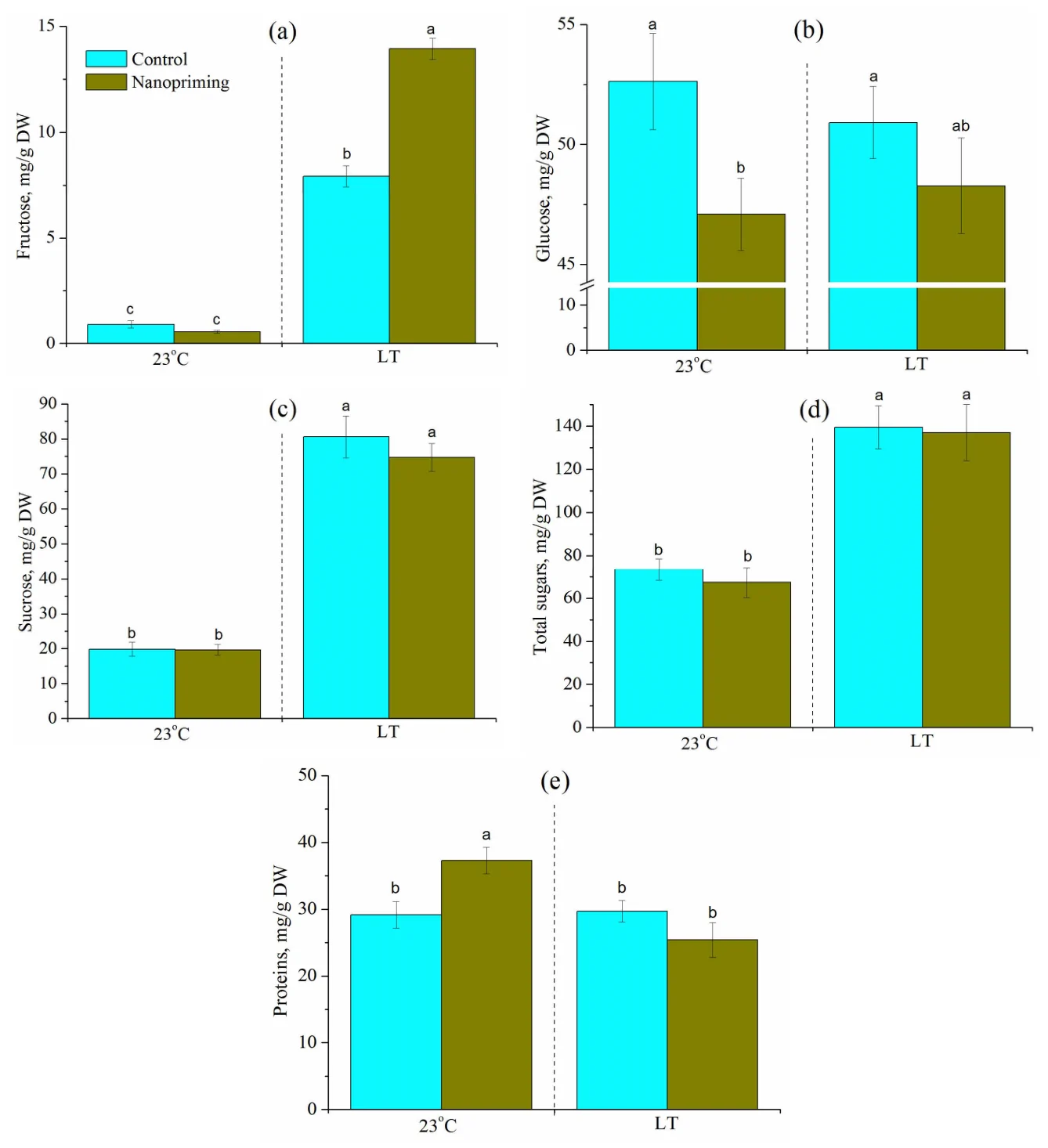
Effect of seed priming with PHF (0.1 mg/L) on the content of fructose (**a**), glucose (**b**), sucrose (**c**), total soluble sugars (**d**) and soluble proteins (**e**) in leaves of wheat seedlings in control and low-temperature conditions. LT—exposure at 4 °C for 5 days. Values that differ significantly at *p* < 0.05 (Tukey’s test) are indicated by different letters. Three biological replicates and three technical assays for each biological repetition were analyzed.

The data on sugar content in leaves at optimal and LT conditions are presented in Figure 4.

At 23 °C, glucose predominated in the leaves, and its content in untreated plants was 11% higher than in the PHF-primed variant (Figure 4b). At the same time, there were no differences between variants in fructose, sucrose and the total sugar content, although in both variants fructose level was minor (less than 1.0 mg/g dry weight) (Figure 4a,c,d).

LT promoted sugar accumulation: the total sugar content in leaves doubled in both untreated and PHF-treated seedlings (Figure 4d). This accumulation was mainly due to a fourfold increase in sucrose content, while glucose content did not differ between variants.

Under optimal temperature, the soluble protein content in leaves of PHF-primed seedlings was 28% higher than in the control (Figure 4e). In LT conditions, protein content in untreated plants remained unchanged, whereas in primed seedlings, it declined to the control level.

### 2.7. Effect of Seed Priming with PHF on the Transcript Levels of Chloroplast-Associated Genes in Leaves of Wheat in Control and Low-Temperature Conditions

**Figure 5:**
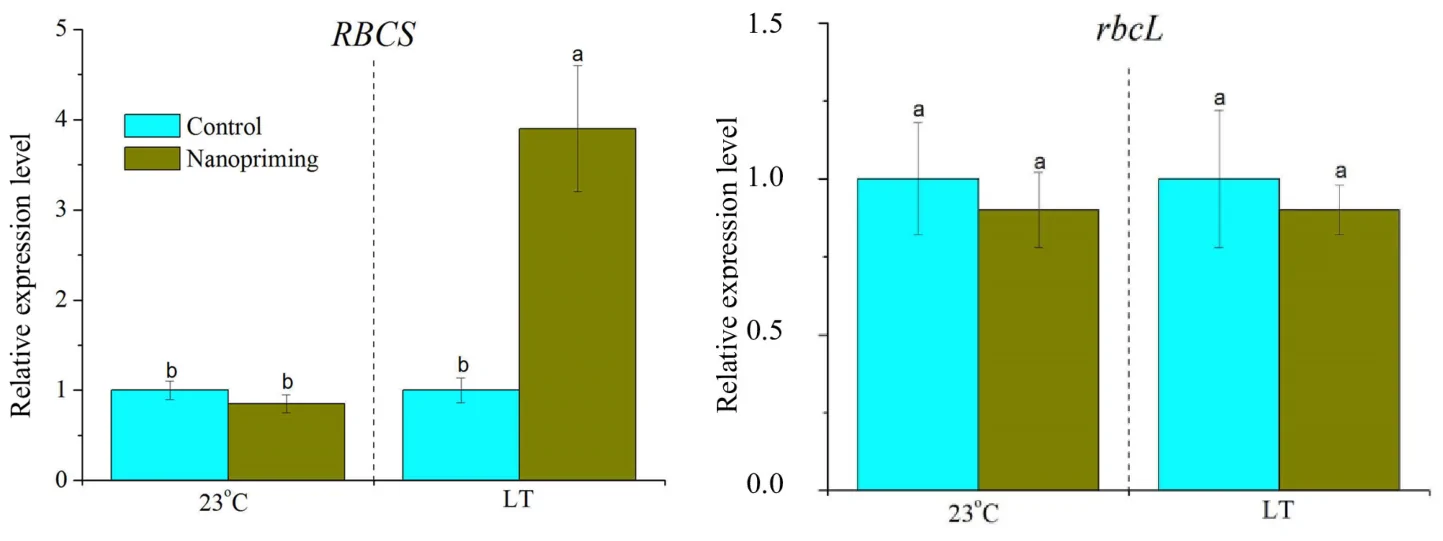

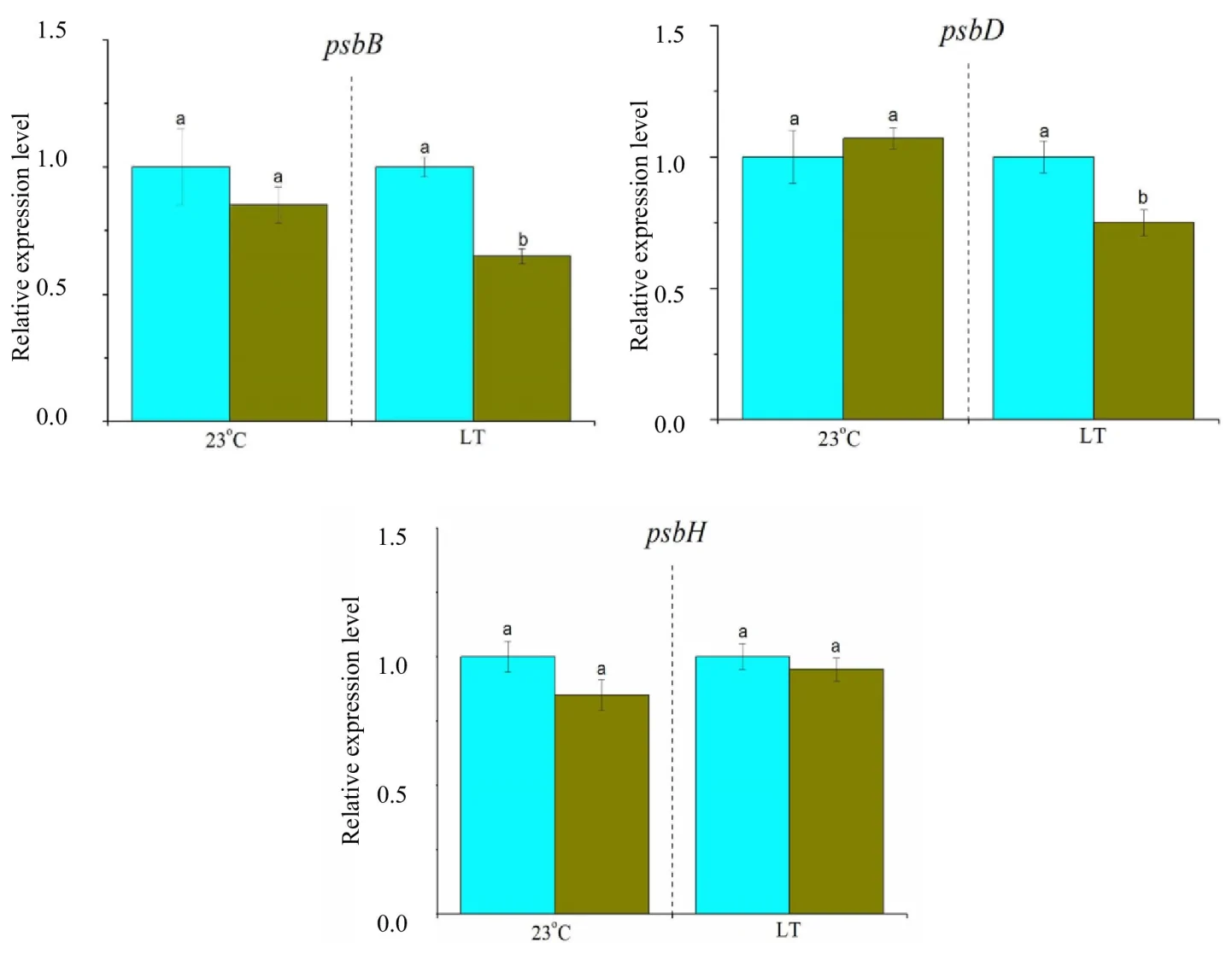
Effect of seed priming with PHF (0.1 mg/L) on the transcript level of chloroplast-associated genes (*RBCS*, *rbcL*, *psbB*, *psbH*, and *psbD*) in leaves of wheat under control and low-temperature conditions. Relative expression level (fold change) was quantified by RT-qPCR, normalized to the reference gene *RP15*, and calculated using the Pfaffl method, with the control sample set to 1. Values that differ significantly at *p* < 0.05 (Tukey’s test) are indicated by different letters. Three biological replicates and three technical assays for each biological repetition were analyzed. LT—exposure at 4 °C for 5 days.

Figure 5 shows the effect of seed priming with PHF on the transcript levels of genes encoding the small (*RBCS*) and large (*rbcL*) subunits of ribulose-1,5-bisphosphate carboxylase/oxygenase (RuBisCO), as well as the genes (*psbB*, *psbD*, *psbH*) encoding core proteins of PSII, in leaves of wheat seedlings under optimal conditions and LT.

Under optimal conditions, primed and control plants did not differ in the expression level of the RuBisCO genes—*RBCS* and *rbcL* (Figure 5). However, after LT, the transcript level of the gene encoding the small RuBisCO subunit (*RBCS*) was increased fourfold in the nanoprimed variant, while no differences between variants were observed in *rbcL* transcript level.

At 23 °C, no differences were detected between variants in *psbB*, *psbH* and *psbD* transcript levels. In LT conditions, transcript levels of *psbB* and *psbD* in nanoprimed seedlings were 30% and 25% lower, respectively, compared with the control, while *psbH* transcript level was not changed.

### 2.8. Effect of Seed Priming with PHF on Pro-/Antioxidant Balance in Wheat Leaves in Control and Low-Temperature Conditions

**Figure 6:**
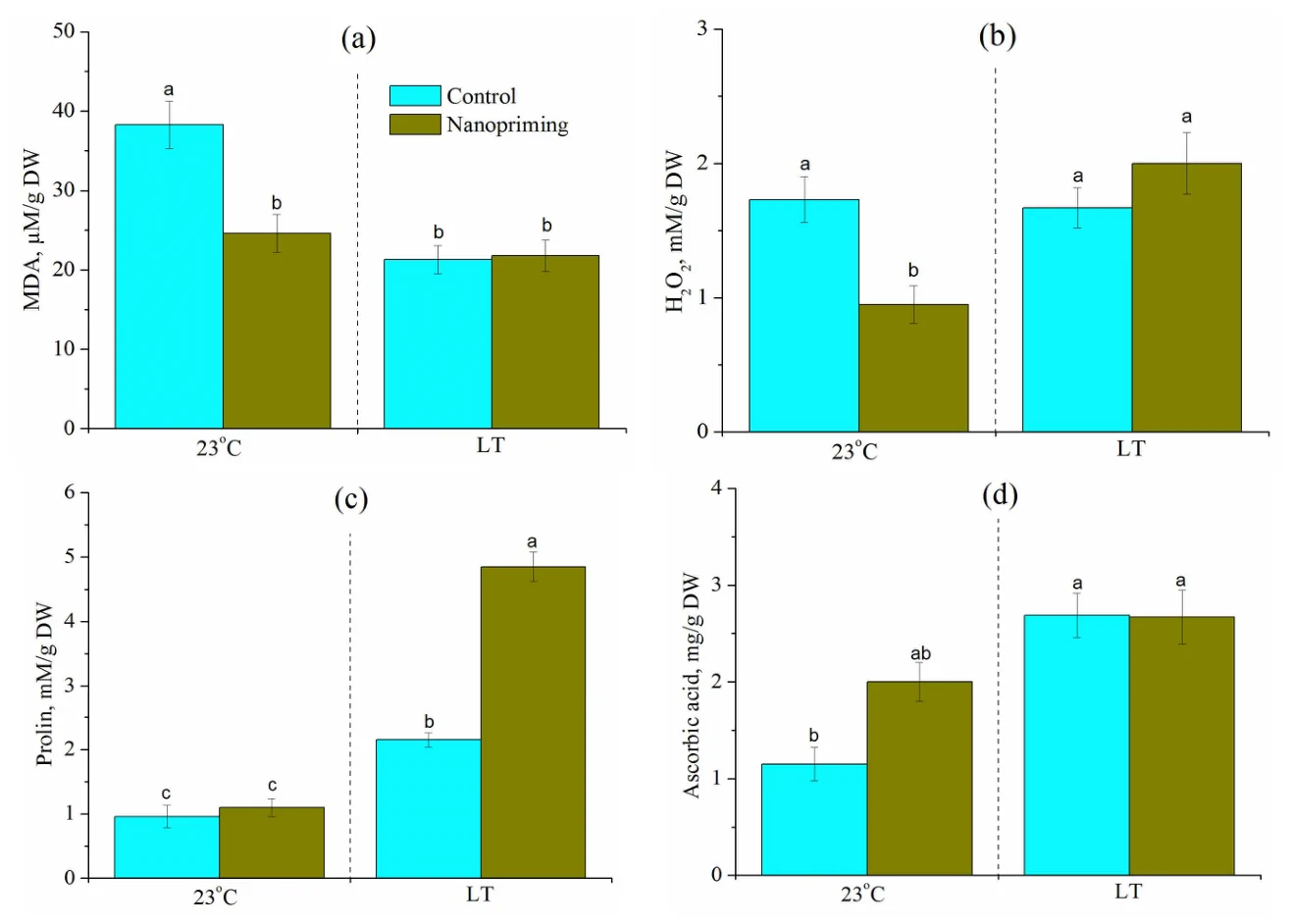
Effect of seed priming with PHF (0.1 mg/L) on the contents of MDA (TBARS) (**a**), H_2_O_2_ (**b**), proline (**c**) and ascorbic acid (**d**) in leaves of wheat in control and low-temperature conditions. LT—exposure at 4 °C for 5 days. Values that differ significantly at *p* < 0.05 (Tukey’s test) are indicated by different letters. Three biological replicates and three technical assays for each biological repetition were analyzed.

We assessed how seed nanopriming with PHF affects the pro-/antioxidant status of wheat leaves by measuring markers of oxidative stress—MDA (thiobarbituric acid reactive substances—TBARS) and H_2_O_2_, key low-molecular-weight antioxidants (proline, ascorbic acid), and the activities of antioxidant enzymes (SOD, POX, CAT, GR). Under optimal conditions, MDA (TBARS) and H_2_O_2_ contents were 35% and 45% lower, respectively, in leaves of seedlings grown from PHF-primed seeds (Figure 6a,b).

Under LT, untreated and primed seedlings did not differ in the content of MDA (TBARS) and H_2_O_2_ in the leaves (Figure 6a,b).

Proline content in leaves of plants from both variants under optimal conditions was identical (Figure 6c). Under LT, proline accumulated in the leaves but to a greater extent in the nanoprimed variant (Figure 6c). In optimal conditions, the ascorbic acid content in leaves of PHF-primed plants was slightly higher than in the control (Figure 6d). After LT exposure, there was a tendency toward ascorbic acid accumulation in leaves, and its content became similar in untreated and primed plants.

**Figure 7:**
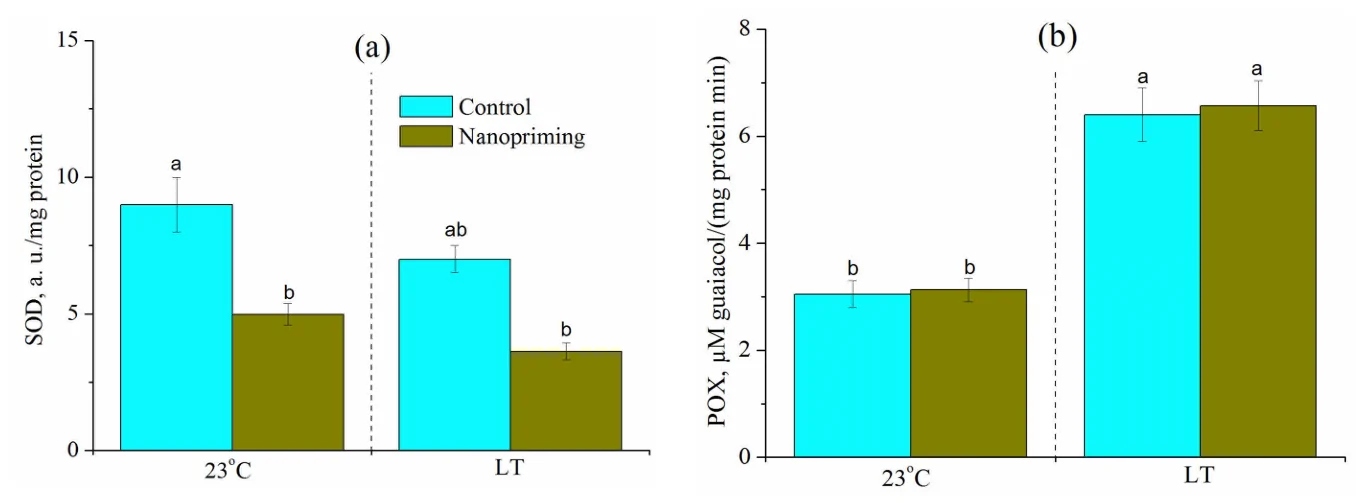

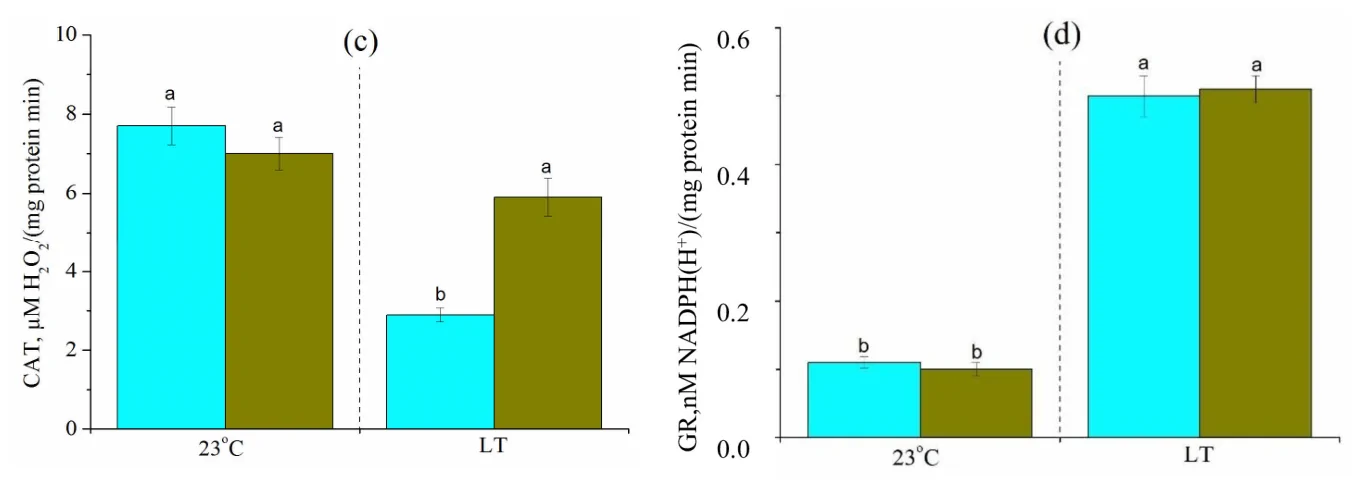
Effect of seed priming with PHF (0.1 mg/L) on the activities of antioxidant enzymes SOD (**a**), POX (**b**), CAT (**c**) and GR (**d**) in leaves of wheat in control and low-temperature conditions. SOD—superoxide dismutase; POX—guaiacol peroxidase; CAT—catalase; GR—glutathione reductase. LT—exposure at 4 °C for 5 days. Values that differ significantly at *p* < 0.05 (Tukey’s test) are indicated by different letters. Three biological replicates and three technical assays for each biological repetition were analyzed.

Leaves of plants from both variants did not differ in POX, CAT and GR activities in optimal conditions. However, SOD activity was 45% lower in leaves of PHF-primed plants than in the control (Figure 7). After LT, POX and GR activities in both variants were increased twofold and fivefold, respectively, while SOD activity remained unchanged (Figure 7a,b,d). In contrast, CAT activity in untreated plants was decreased 2.5-fold under LT, whereas in primed plants it did not change (Figure 7c).

### 2.9. Effect of Seed Priming with PHF on Transcript Level of COR Genes in Leaves of Wheat in Control and Low-Temperature Conditions

**Figure 8:**
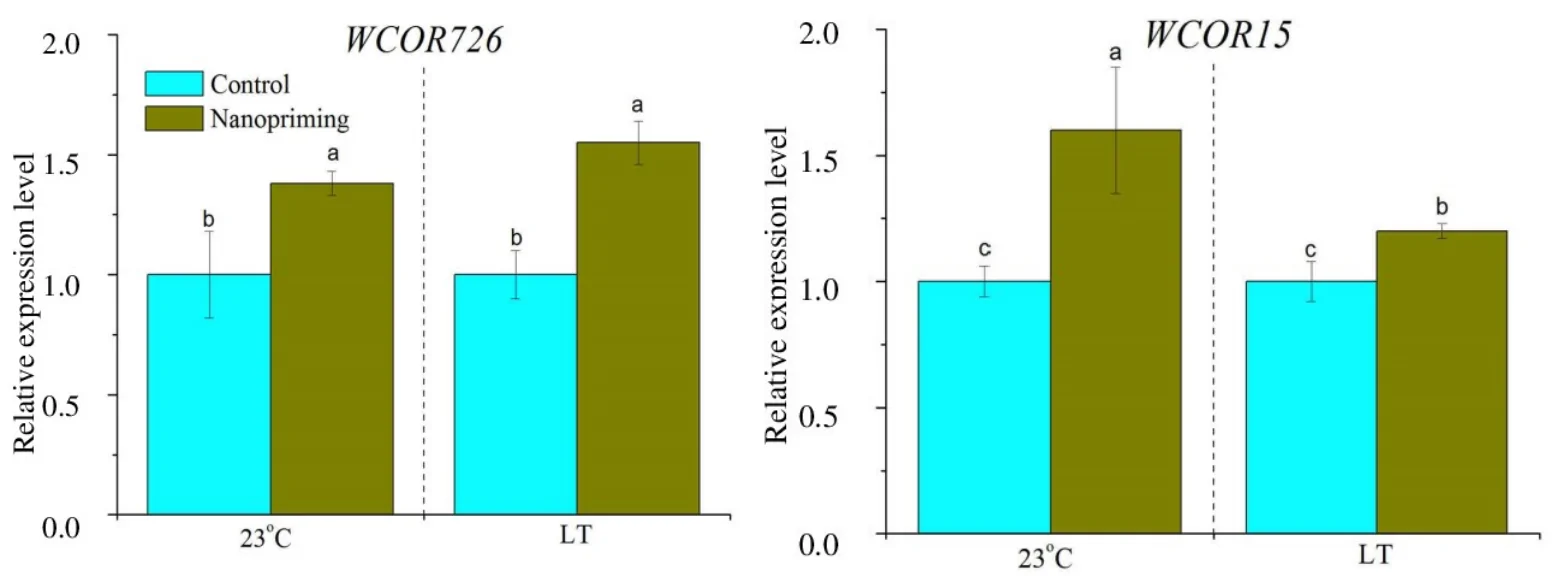
Effect of seed priming with PHF (0.1 mg/L) on the transcript level of COR genes (*WCOR15* and *WCOR726*) in leaves of wheat under control and low-temperature conditions. Relative expression level (fold change) was quantified by RT-qPCR, normalized to the reference gene *RP15*, and calculated using the Pfaffl method, with the control sample set to 1. Values that differ significantly at *p* < 0.05 (Tukey’s test) are indicated by different letters. Three biological replicates and three technical assays for each biological repetition were analyzed. LT—exposure at 4 °C for 5 days.

Figure 8 shows the effect of seed priming with PHF on the transcript level of cold-responsive genes (*WCOR15*, *WCOR726*) in leaves of wheat seedlings under optimal conditions and LT. In optimal conditions, transcript levels of *WCOR726* and *WCOR15* were almost 1.5-fold higher in PHF-primed plants than in the control. After LT treatment, transcript levels of these COR genes remained higher in the nanoprimed plants.

### 2.10. Identification of Correlation Dependencies and Hierarchical Clustering of Data

**Figure 9:**
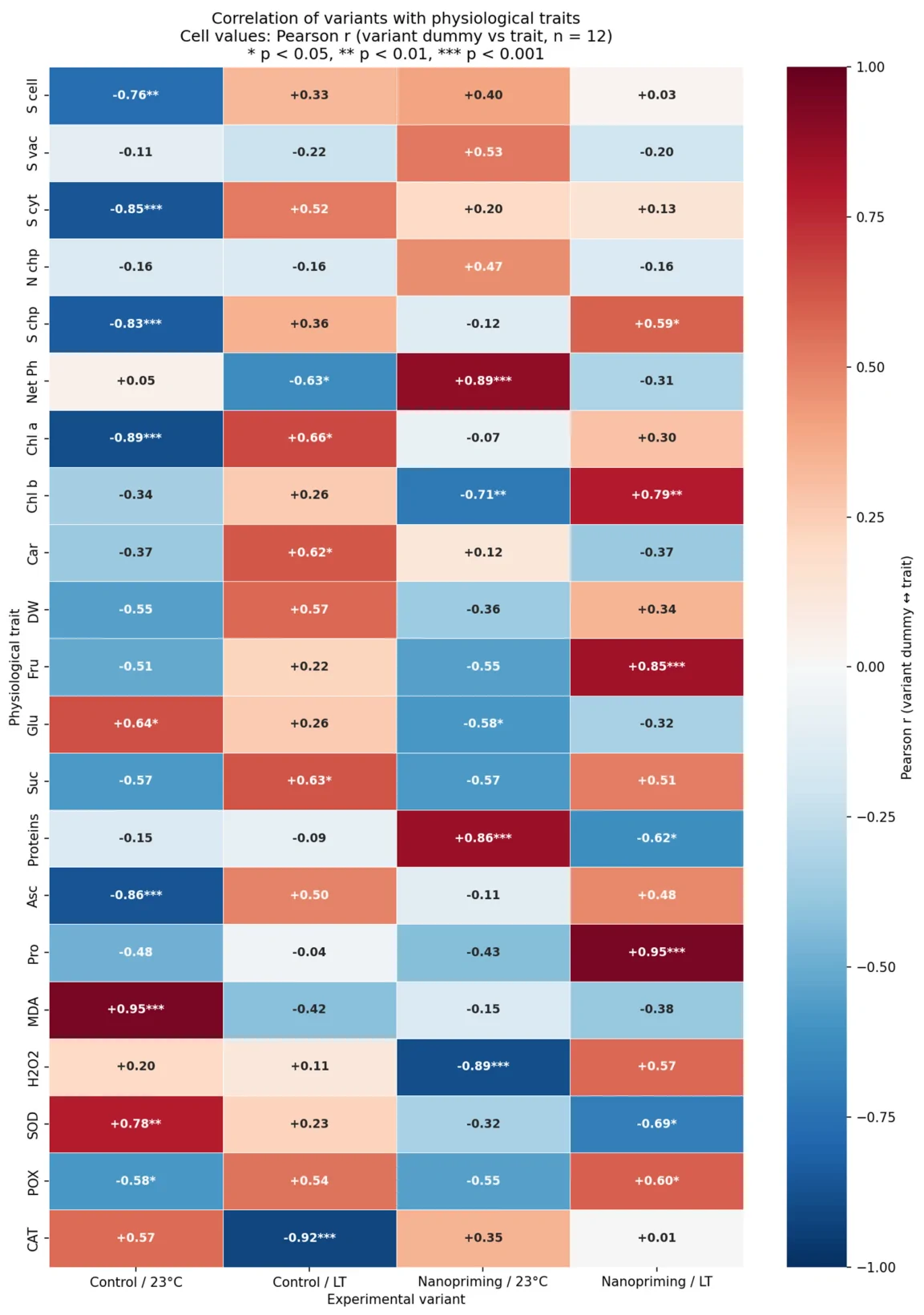
Correlation analysis between experimental variants and physiological parameters.

Correlation analysis between experimental variants and 22 physiological indicators revealed a number of highly significant associations (*p* < 0.001), which can be grouped into four thematic blocks (Figure 9). These associations are: (1) markers of oxidative stress; (2) osmolytes and cold acclimation; (3) photosynthetic apparatus; and (4) morphometric parameters and protein content. It has been experimentally confirmed that nanopriming reduced accumulation of ROS under optimal conditions. In the Nanopriming/LT variant, the proline (Pro) content was maximal (r = +0.95; *p* < 0.001), similar to the fructose (Fru) content (r = +0.85; *p* < 0.001). The accumulation of Pro and Fru under the combined action of nanopriming and LT may indicate a synergistic effect. Sucrose (Suc) content was increased in both variants at LT (r ≈ +0.6), which is consistent with the protective role of sucrose. The photosynthetic intensity (Net Ph) had the highest positive correlation in the Nanopriming/23 °C variant (r = +0.89; *p* < 0.001), while chloroplast area (S chp) was greatest in the Nanopriming/LT variant (r = +0.59; *p* < 0.05). The content of chlorophyll *b* was maximal in the Nanopriming/LT variant (r = +0.79; *p* < 0.01), while the content of chlorophyll *a* was minimal in the Control/23 °C (r = −0.89; *p* < 0.001). These data indicate that nanopriming with PHF stabilized the photosynthetic apparatus in LT conditions. The cytoplasm area (S cyt), cell area (S cell), and chloroplast area (S chp) were minimal in the Control/23 °C variant (r = −0.85–−0.83; *p* < 0.001), and maximal in the Control/LT and Nanopriming variants/23 °C. This indicated the influence of nanopriming on structural parameters.

**Figure 10:**
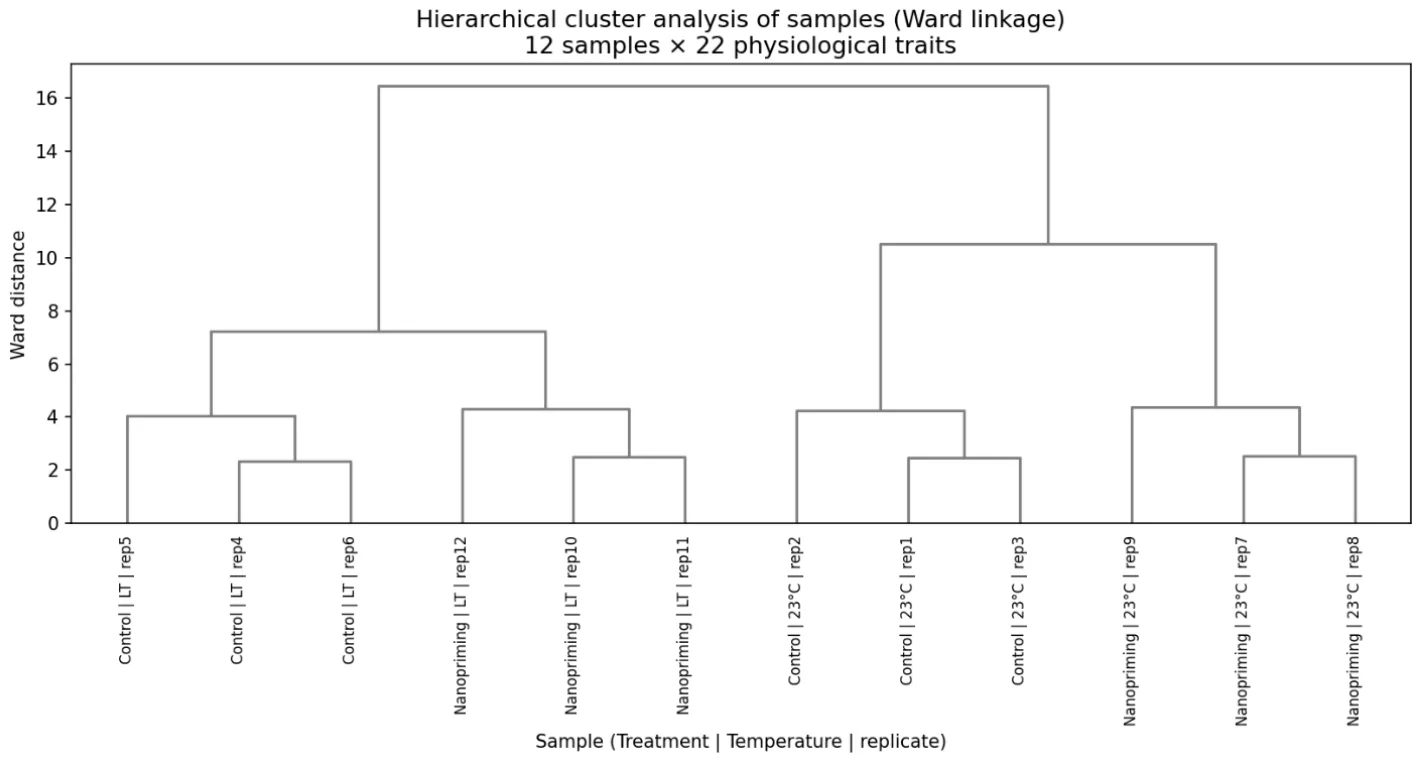
Hierarchical clustering by Ward’s algorithm, Euclidean distance according to Z-standardized features of 12 separate groups. Each dendrogram sheet is signed with a combination of factors and a biological replication number.

Figure 10 shows that at the top level of the hierarchy (Ward distance ≈16), the studied variants are divided into two separate superclusters. The left supercluster unites six groups at the LT point. The right supercluster unites six groups at 23 °C. The first branch of the dendrogram corresponds to the temperature factor, which indicates its dominant influence in the experiment. At the next level of clustering (the distance between points ≈7 and ≈11), each temperature supercluster is divided into two subclusters. In the LT supercluster, the Control/LT plants form their own subcluster, to which the primed plants join at a short distance. In the 23 °C supercluster, the three Control groups/23 °C and the three Nanoprimed groups/23 °C form well-defined subclusters. All three biological groups of each experimental variant are combined at minimum distances between cells (~2–4). This confirms the high reproducibility of physiological and biochemical changes within the variant.

**Figure 11:**
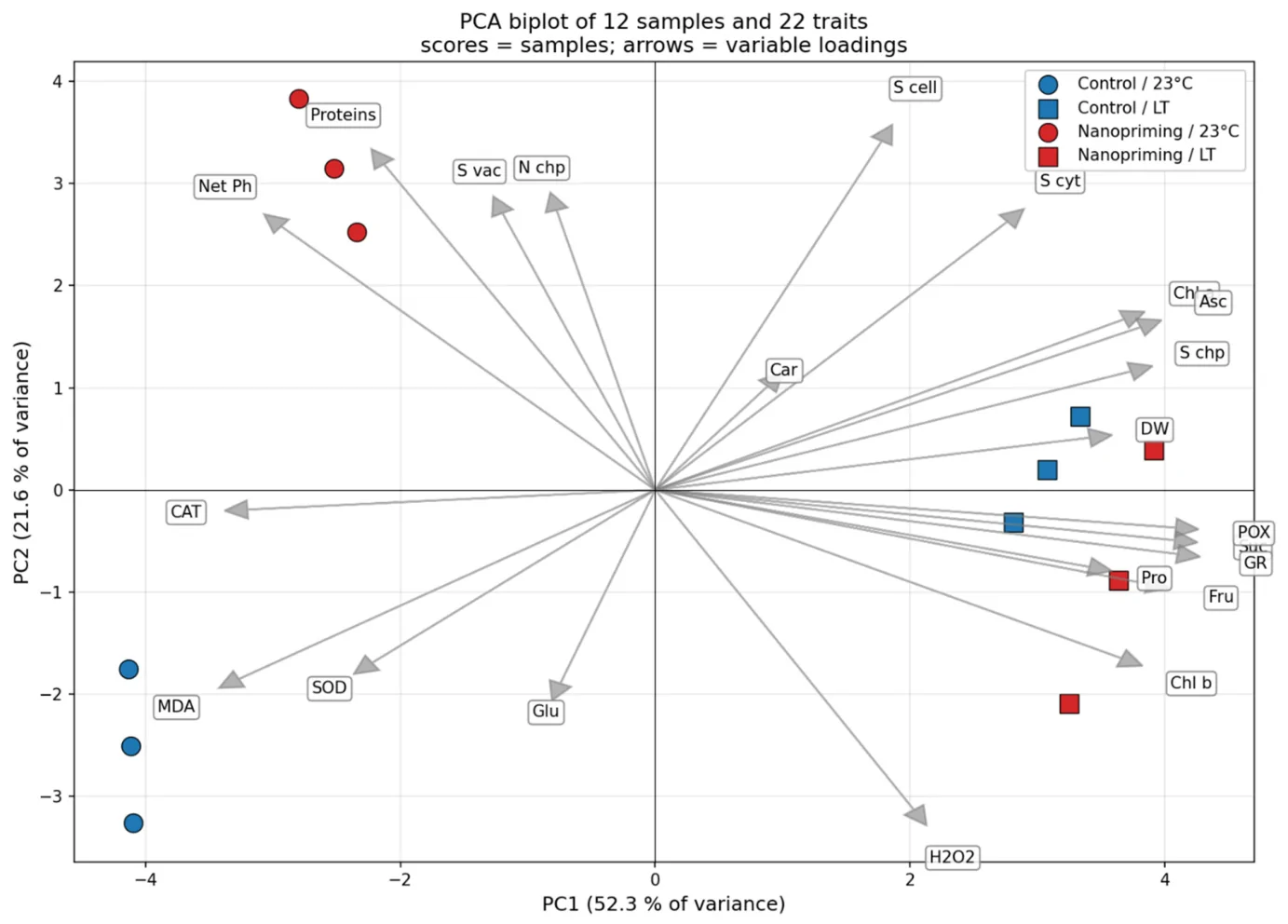
PCA biplot on 22 morphophysiological and biochemical parameters of wheat seedlings. The indicators of individual plants are projected onto the plane formed by the first and second main components, which, together, describe 73.9% of the total variance (PC1—52.3%, PC2—21.6%). The arrows indicate loads (projections) of variables scaled by the square root of the corresponding eigenvalue; only the direction of the arrows and their relative length have biological meaning.

Thus, based on the dendrogram, two conclusions can be drawn. Firstly, LT is the dominant factor organizing the physiological and biochemical variability in this experiment. Secondly, nanopriming has a measurable and reproducible effect within each temperature regime. This is evidenced by the stable subcluster division of plants. Nanopriming narrows, but does not completely eliminate, the physiological gap induced by LT, which is consistent with the results of the principal component analysis (Figure 11).

Principal component analysis (PCA) was used to reduce the complexity of multidimensional data as a way to identify patterns and present data in a way that highlights similarities and differences. Four experimental groups occupied separate, non-overlapping regions on the PC1–PC2 plane (Figure 11), indicating that nanopriming and/or LT form reproducible and distinguishable phenotypes of wheat seedlings.

The first main component (PC1) describes the main stress-protection axis, while the second (PC2) reflects morphological variability. Thus, PC1 and PC2 describe the two main axes of the wheat germ reaction: (1) the “stress protection” axis, along which nanopriming effectively counteracts the biochemical effects of LT, and (2) the “morphological” axis, along which nanopriming has a strong, largely independent effect on LT on cell size and protein accumulation.

## 3. Discussion

### 3.1. Effect of Seed Priming with PHF on Wheat in Control Conditions

The present study was conducted using the carbon nanomaterial PHF [C_60_(OH)_24_]. This molecule has a diameter of around 1.0 nm with symmetrically arranged hydroxyl groups on the C_60_ sphere [9]. It can be dissolved in water in concentrations ranging from 10^−3^ mol/L to 5 × 10^−3^ mol/L, and in the pH interval from 3.5 to 9.8 [35]. In aqueous solutions, PHF molecules interact with each other, as well as with water molecules. They form polydisperse suspensions consisting of stable negatively charged nanoparticles [10,36]. Hydrogen atoms in hydroxyl groups interact with water molecules through hydrogen bonds and van der Waals interactions [37,38]. According to our DLS data, the average electrokinetic potential of PHF solutions was −32.5 mV, indicating the electrostatic stability of the colloidal solution. The negative charge of the PHF solution is explained by bond stretching or deprotonation of hydroxyl groups of fullerenol particles. Therefore, PHF solution may be a suitable carrier system for practical use in biotechnology [39,40,41]. PHF [C_60_(OH)_24_] has demonstrated stability and reproducibility in biological research due to its hydrophilicity and commercial availability [42,43].

In our study, for the first time, the effect of pre-sowing treatment of wheat seeds with PHF [C_60_(OH)_24_] on the physiological and biochemical parameters of seedlings under control and LT conditions was studied. The choice of seed nanopriming in comparison with root and foliar treatment is justified by the greater economy and environmental benefits of this method of plant treatment [24,44]. During concentration tests, we selected the minimum concentration of PHF solution—0.1 mg/L—which had a stimulating effect on germination. The positive effect of PHF on seed germination has also been noted by other authors. Such effects were found on bitter melon [17], *B. napus* [21], and *Triticum aestivum* [24]. It can be assumed that the stimulating effect of PHF on seed germination energy is due to better water absorption and the process of seed swelling. Studies show the ability of nanoparticles to enhance the ability of seeds to absorb water [45]. The nanoscale size of carbon-based nanomaterials allows them to penetrate the seed coat or cell wall, potentially creating new pores or activating aquaporins [5]. Priming induced an increase in the activity of enzymes such as amylases, proteases, and lipases that break down macromolecules for growth and development of the embryo [46].

One of the ways to determine the effect of nanoparticles on plants is by measuring the root length [47]. An analysis of the growth parameters of wheat seedlings showed the stimulating effect of PHF on the length of the axial organs—root and leaf. A similar positive effect of nanopriming with various PHF on growth processes was found in sugar beet seedlings [22], cabbage [21,48], and wheat [24]. A possible mechanism for accelerating root growth is associated with PHF stimulation of the process of longitudinal stretching of cells in the growth zone [49]. It was shown that PHF solution affects the conformation and function of membranes [50]. In this case, PHF treatment reduces the distance between lipid molecules near the adsorption site, thereby increasing the fluidity of the outer layer. PHF molecules can also penetrate deep into the membrane and increase the fluidity of the hydrophobic region. PHF is involved in the modulation of membrane ATPases and, as a result, in the transport of potassium ions across membranes [51].

A key biometric indicator of seedling viability is its dry mass. Our experiments showed that seedlings grown from primed seeds had a larger biomass compared to the control. This is quite expected, since an increase in the growth rate affects the growth of their biomass.

Under the influence of nanopriming with PHF, the area of cells, cytoplasm, and chloroplasts was increased compared with the control. It should be noted that gold nanoparticles had a similar effect on the ultrastructure of chloroplasts [52]. The authors noted that under the influence of nanopriming, the area of chloroplasts and the number of starch grains were increased. At the same time, the chloroplasts became more rounded and had a denser stroma. The structural changes observed in plants are consistent with changes in the physiological and biochemical parameters of PSA. After nanopriming with PHF, the seedlings had a higher photosynthetic intensity, which was accompanied by an increased content of chlorophyll *a* and a higher ratio of chlorophyll *a*/*b*. A high value of the chlorophyll *a*/*b* ratio indicates a low degree of thylakoid stacking, i.e., a small proportion of appressed thylakoid membranes [53]. In addition, Chl *a*/*b* is an indicator of plant acclimation to light intensity [54]. Therefore, it can be concluded that PSA of primed plants is more adapted to light conditions. Our data are consistent with the results of other studies showing that PHF improves chlorophyll synthesis [55] and gas exchange in wheat [26]. The increased content of chlorophyll *a* in the leaves of PHF-primed seedlings can provide higher physiological activity of PSA. The results of our research indicate a stable level of transcripts of PSA-associated genes (*psbB*, *psbH*, *psbD*) encoding transmembrane chlorophyll-binding proteins of PSII. It can be assumed that the increased photosynthesis is due to the stimulating effect of PHF on the level of thylakoid proteins involved in electron transfer, as was shown in another study [40].

Note that there were no significant differences between the variants in terms of the total content of soluble sugars. The low content of free fructose in the leaves of seedlings of both variants is probably due to its rapid involvement in metabolism by entering into a phosphorylation reaction. At the same time, the glucose content in the leaves of seedlings grown from primed seeds was 11% lower. A comparative analysis of the data on growth indicators and sugar content suggests a more active involvement of glucose in the metabolic processes associated with the development of axial organs in PHF-primed seedlings. There is evidence that the expression of enzymes catalyzing the steps of glycolysis was up-regulated by PHF [40].

The structural changes in cells and chloroplasts that occurred in PHF-primed seedlings were accompanied by a higher content of osmolytes—soluble proteins, proline and ascorbic acid. This is consistent with the opinion that the synthesis of many osmolytes is associated with an increase in the size of chloroplasts [56].

Note that the leaves of seedlings grown from PHF-primed seeds contained fewer markers of oxidative stress—H_2_O_2_, MDA (TBARS), and the activity of SOD in the primed plants was lower compared with the control. The ability of PHF to reduce ROS levels was established in maize plants under drought conditions [20] and wheat under salt stress [25]. In a model liposome system that mimics cell membranes, it was shown that treatment of PHF dose-dependently suppresses the formation of MDA [57]. It is believed that PHF treatment exhibits antioxidant properties due to the presence of a delocalized system of π-double bonds on the surface of molecules [9,58,59]. PHF treatment reduced ROS levels and damage caused by POL in wheat under drought conditions [24]. PHF spray did not reduce leaf MDA content although it substantially decreased H_2_O_2_ [26]. PHF spray increased SOD, CAT, POD and APX activities under salt stress [25].

**Figure 12:**
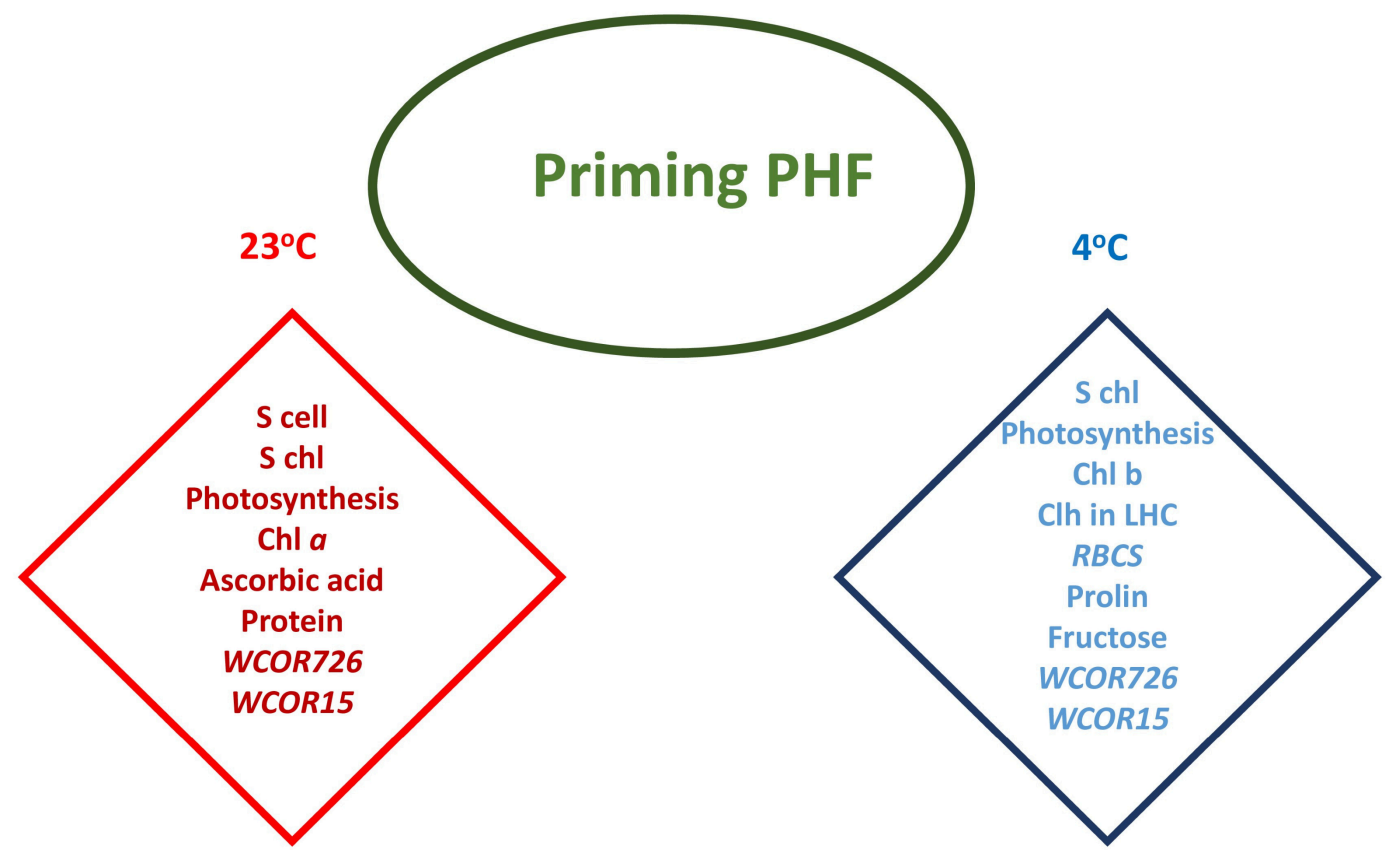
Stimulating effect of priming with PHF on wheat physiological and biochemical parameters under control and low-temperature conditions.

Thus, under control conditions, nanopriming with PHF enhanced the growth processes, which was accompanied by an increase in the size of mesophyll cells, cytoplasm, and chloroplasts. At the same time, priming increased the intensity of photosynthesis and the content of chlorophyll *a*. The decrease in indicators of oxidative stress was accompanied by an increase in protein, ascorbic acid content, and the expression of genes encoding dehydrins (Figure 12). A decrease in SOD levels was noted, while no effect on other antioxidant enzymes was observed. The effect of nanopriming on the balance of pro-/antioxidants is probably more related to the effect on the low-molecular-weight component.

### 3.2. Effect of Seed Priming with PHF on Wheat in Low-Temperature Conditions

For the first time, we studied the effect of PHF seed nanopriming on some physiological and biochemical parameters in wheat seedlings under LT. Previously, we showed that prolonged exposure of wheat seedlings in LT conditions (4 °C) contributed to an increase in their cold tolerance, i.e., the process of cold acclimation took place [60]. Note that acclimation is the capacity to adapt to environmental changes within the lifetime [61]. Cold acclimation comprises reprogramming of the transcriptome, proteome and metabolome and affects communication and signaling between subcellular organelles [62]. The effects of cold acclimation on wheat plants result in the precise reprogramming of the expression of genes, including COR genes as well as genes encoding antioxidative enzymes, late embryogenesis abundant (LEA) proteins, and chaperones [63]. In the process of cold acclimation of plants, the following main adaptive changes are distinguished: modification in membranes, accumulation of compatible osmolytes, low-molecular-weight antioxidants and protective proteins, as well as biochemical changes aimed at maintaining PSA function [64]. In this regard, structural changes in cells and chloroplasts, as the main photosynthetic organelles, occupy an important place in the process of cold acclimation [56].

One of the adaptive structural reactions of plants under LT conditions is an increase the size of mesophyll cells due to an increase in the volume of cytoplasm and an increase in the size of chloroplasts [56]. This reaction is observed in primed plants already under control conditions. Such a structural restructuring indicates the redistribution of water and substances with protective functions in plants.

An important functional indicator of plants after cold acclimation is PSA activity [62]. In our study, it was found that in the cold acclimation process, the photosynthetic intensity was decreased by 2 times, but in the nanopriming variant, it was 50% higher than in non-treated plants. The content of chlorophylls after LT in the leaves of seedlings of both variants was increased, while in the variant with nanopriming, an increase in the proportion of chlorophyll in LHC was observed. Note that primed seedlings contained more chlorophyll *b* than untreated ones. It is known that chlorophyll *b* is an important regulator of LHC biosynthesis and degradation. Chlorophyll *b* is located mainly in the LHC and participates in the transfer of absorbed energy to the reaction center [65]. The increased content of chlorophyll *b* in the chloroplasts of primed wheat seedlings may play an important role in maintaining the intensity of photosynthesis during LT [66]. In addition, the increase in photosynthesis intensity in PHF-primed seedlings after LT may also be due to a fourfold increase in the transcript level of the *RBCS* gene encoding the small subunit of RuBisCO, a key enzyme of the photosynthetic process [67]. It was shown that carbon-modified plants exhibit an improvement in RuBisCO activity [68]. The stimulating effect of pre-sowing PHF seed treatment on photosynthesis is also shown in *B. napus* under water stress [21].

After LT, the dry matter in the leaves of seedlings of both variants increased, which is probably due to cryoprotective dehydration [69]. An increase in the dry weight during cold acclimation is associated with the accumulation of protective substances (soluble sugars, ascorbic acid and proline). Note that the accumulation of soluble sugars and ascorbic acid occurred under the influence of LT in both untreated and nanoprimed plants. The accumulation of sugars plays an important role in the process of plant adaptation to cold [70]. It is known that the functions of free sugars are to stabilize and protect the structure of membranes, lipid molecules, and proteins from cold damage, neutralize ROS, and participate in signaling and metabolic processes as an energy source and precursors for the synthesis of other protective compounds [71,72]. The important role of ascorbic acid as a non-enzymatic antioxidant in the neutralization of ROS (primarily H_2_O_2_) is well known [73]. Our studies showed that after LT, the seedlings treated with PHF had a higher proline content in the leaves (4.5 times). Proline is known to play an important protective role in plants under suboptimal conditions, when it acts as a molecular chaperone protecting protein integrity, a membrane stabilizer, a signaling molecule, an inducer of osmotic genes, and an interceptor of ROS radicals [64,74,75,76]. During foliar treatment of wheat plants with PHF, accumulation of proline in leaves was also observed both under salinization and without it [26].

It is known that PHF are able to enhance non-enzymatic antioxidant activity, possibly due to the presence of hydroxyl groups on their surface, which form hydrogen bonds between the PHF molecule and various biological compounds [28,77]. Under LT conditions, SOD activity in PHF-primed seedlings was lower than in the control, which is probably related to the ability of fullerene to mimic the action of SOD [39,78]. Mn_3_O_4_ nanoparticles have also been shown to possess multiple enzyme-mimicking activities [79,80]. Cerium oxide nanoparticles exhibit catalase mimetic activity [81]. It is important to note that under the action of abiotic stress factors, PHF treatment activated the enzymes of the antioxidant system, which was shown in wheat [48], sugar beet [22], maize [20] and rapeseed [21].

There is a positive correlation between the expression level of COR genes and plant cold tolerance [82,83]. It is known that the *WCOR726* and *WCOR15* genes encode LEA dehydrin proteins, which perform protective functions in plant cells under stress [84]. At the same time, the protein encoded by *WCOR15* is localized in chloroplasts, where it protects PSII [85]. The results of our studies indicate that the relative level of *WCOR15* and *WCOR726* transcripts in the leaves of PHF-primed seedlings was higher than in the control and at low temperature. One of the central mechanisms of genetic control of cold tolerance is the ICE-CBF-COR pathway. Our studies showed an increase in the level of *WCOR15* and *WCOR726* transcripts in primed plants already under control conditions. Perhaps this primary change causes an increase in the synthesis of protective molecules (soluble proteins, sugars, proline, ascorbic acid, carotenoids) in wheat under control conditions. This helps to increase the cold tolerance of wheat in the future at low temperatures. It can be assumed that fullerenol acts not only as an antioxidant, but also as a trigger for the synthesis of additional protective compounds.

Thus, under LT conditions, PHF nanopriming enhances photosynthesis intensity by increasing chloroplast area, content of chlorophyll *b* and the proportion of chlorophyll in LHC, as well as increasing content of proline, *RBCS* and COR-gene expression (Figure 12).

## 4. Materials and Methods

Characterization of PHF [C_60_(OH)_24_]. PHF [C_60_(OH)_24_] with mass fraction purity 99.8% produced in “MST Nano” (St. Petersburg, Russian Federation) was applied for study. PHF [C_60_(OH)_24_] has been previously analyzed using various physicochemical methods, such as IR, UV/Vis spectroscopy, NMR spectroscopy, mass spectrometry, and elemental analysis [86]. Data on the temperature dependence of solubility in water, concentration dependence of density, hydrogen ion concentration, molar conductivity, dissociation constant, and dynamic light scattering were published earlier [87].

In this work, we determined the electrokinetic potential (ζ-potential) and particle diameter of a colloidal fullerenol solution using a Litesizer 500 laser analyzer (Anton Paar, Graz, Austria) using dynamic light scattering (DLS). The method is based on a combination of laser Doppler velocimetry and laser light scattering to analyze the electrophoretic mobility of dispersion particles and then calculate their ζ-potential using the Henry equation:

ζ = *f* (ηU_E_/ε ε_0_),

where ζ—electrokinetic potential, *f*—numerical coefficient depending on the shape of the particles and the ratio of their size to the diffuse part of the double electric layer, η—viscosity of the medium, U_E_ —electrophoretic mobility of particles, ε—dielectric constant of the medium, and ε_0_—dielectric constant. When an electric field is applied, charged particles are attracted to an oppositely charged electrode. At the same time, their speed of movement and electrophoretic mobility depend on the particle charge, viscosity, and dielectric constant of the medium.

To determine the characteristics of the particles, 0.75 mL of the test sample was placed in a polystyrene cuvette “DTS1060” using a syringe. At least 3 measurements were carried out at a temperature of 25 °C; from the results of each series, the average value was determined.

Fullerenol application, seedling growth and low temperature treatment. Wheat seeds (*Triticum aestivum* L., Poaceae) of the cold-sustainable genotype Zlata (Federal Research Center “Nemchinovka”, Moscow, Russia) were used in the experiments. Seed priming was performed using PHF [C_60_(OH)_24_] solutions at concentrations of 0.05, 0.1, 1, 10, 20 and 30 mg/L for 24 h. The control seeds were treated with distilled water. The seeds (100 seeds for each variant) were placed in Petri dishes and about 20 mL of PHF solution was added to each. Petri dishes were kept for 24 h in a chamber at 23 °C in the dark. Next, seeds were germinated in distilled water at a temperature of 23 °C, relative humidity 60–70%, and illumination 100 µmol photons/(m^2^ s) with a photoperiod of 16 h. As the water evaporated, distilled water was periodically added to the seedling container. During the period of seedling growth, no nutrients were added to the distilled water, and seedling development occurred only due to the nutrients in the seed endosperm. The germination of seeds was analyzed as follows: seed germination energy (%) = the number of germinated seeds on 3 days × 100/the number of seeds; seed germination (%) = the number of germinated seeds on 7 days × 100/the number of seeds [20]. 35-45 wheat seedlings aged 14 days were randomly selected to determine the mass, length of the leaf and root.

After reaching 10 days of age, some of the seedlings were transferred to a KBW-240 climatic chamber (Binder, Tuttlingen, Germany) at 4 °C for 5 days, keeping other conditions unchanged. The choice of low temperature for adaptation (4 °C) is based on previous research [60,88]. In these studies, we showed that tolerance to low temperature increased after adaptation to 4 °C. This conclusion was made based on data on the survival percentage of control and low-temperature-adapted plants at 4 °C after test freezing.

Leaf dry weight (DW) of seedlings was determined after drying in a thermostat at 100 ± 5 °C to constant weight and expressed as a percentage of the initial fresh weight.

Structure of leaf mesophyll cells. For microscopy, the samples from the middle parts of 3–4 leaves were fixed via the standard method for 4 h in 2.5% glutaraldehyde in 0.1 M phosphate buffer (pH 7.4) at 0–4 °C with postfixation in 2% OsO_4_. The samples were dehydrated in an ethanol series (20–100%), and the material was embedded in “Epon-812”. Slices of leaves were prepared via an LKB-3 microtome (LKB, Sollentuna, Sweden). The slices were observed under an “AxioImager D1” (Carl Zeiss, Oberkohen, Germany). Morphometric analysis of the structures of the cells in the first subepidermal layer of the mesophyll was performed via the program “Axion Vision 4.8”.

Net photosynthesis and photosynthetic pigments. The concentrations of chlorophyll *a* (Chl *a*), chlorophyll *b* (Chl *b*), and total carotenoids (Car) were determined spectrophotometrically (Genesys 10UV spectrophotometer, Thermo Electron Corporation, Waltham, MA, USA) at wavelengths corresponding to the absorption maxima of Chl *a*, Chl *b* and Car in 80% acetone—663 nm, 646 nm and 470 nm, respectively. Pigment concentrations were calculated using the following formulae [89]: Ca = 12.21D_663_ − 2.81D_646_, Cb = 20.13D_646_ − 5.03D_663_, and Ccar = (1000D_470_ − 3.27Ca − 104Cb)/198. The pigment content was expressed in mg/g DW of leaves. We calculated the chlorophyll portion in LHC, assuming that practically all Chl *b* was located in LHC and the chlorophyll ratio in this complex was equal to 1.2 [90].

Net photosynthesis was measured in an open-type unit equipped with a URAS-2T infrared gas analyser (Hartmann und Braun, Heidenheim, Germany) [91].

Soluble sugars and proteins. To determine the content of soluble sugars (glucose, fructose, sucrose), weighed samples (500 mg) of leaves were fixed with 96% boiling ethanol. Tissue was homogenized and sugars were extracted with 80% ethanol three times. In the obtained extracts, the content of fructose was determined according to Roe, followed by conversion to sucrose content [92]. The glucose content was determined by the glucose oxidase method using the Olvex diagnosticum Kit (Vital Diagnostics, Moscow, Russia).

To determine the content of soluble proteins, leaf samples (500 mg) were homogenized in isolation medium (50 mmol Tris-HCl, pH 7.6, 3 mmol EDTA, 250 mmol sucrose, 3.6 mmol cysteine, 5 mmol ascorbic acid, 3 mmol MgCl2, 2 mmol DTT, 2 mmol PMSF), and centrifuged for 20 min at 16,000× *g*. The isolation medium for glutathione reductase additionally contained 2% polyvinylpyrrolidone and 0.2% Triton X-100. The extract was purified on a “PD-10 midiTrap G-25” column (GE Healthcare, Chicago, IL, USA). The protein content in the supernatant was determined spectrophotometrically at 562 nm using the “Bicinchoninic Acid Kit” for Protein Determination (BCA1-1KT, Sigma-Aldrich, Milwaukee, WI, USA), according to the manufacturer’s protocol.

RT-qPCR. Total RNA was isolated from leaf samples (50 mg) using the Spectrum Plant Total RNA Kit (Sigma-Aldrich, St. Louis, MO, USA). RNA quality and integrity were assessed by NanoDrop-2000 spectrophotometry (Thermo Fisher Scientific, Waltham, MA, USA) and agarose gel electrophoresis. Genomic DNA was removed with DNase I (Thermo Fisher Scientific, Waltham, MA, USA), and cDNA was synthesized using the RevertAid Reverse Transcription Kit (Thermo Fisher Scientific, Waltham, MA, USA). RT-qPCR was performed on a CFX96 Touch system (Bio-Rad, Hercules, CA, USA) using SYBR Green I (Evrogen, Moscow, Russia). Primers for *WCOR726*, *WCOR15*, and *RP15* were designed using online resources Primer-BLAST (https://www.ncbi.nlm.nih.gov/tools/primer-blast/, accessed on 1 February 2026) and OligoAnalyzer™ Tool (https://eu.idtdna.com/pages/tools/oligoanalyzer, accessed on 1 February 2026) (Table 3).

Gene-specific primers (Table 3) for the amplification of large and small subunits of RuBisCo (*RBCS* and *rbcL*) target genes were borrowed from [93] and primers for PSII protein genes (*psbB*, *psbH*, *psbD*) were taken from [94]. The amplification efficiency of the validated primer pairs was determined using standard curves generated from serial dilutions of cDNA. The amplification efficiency ranged from 90 to 110%, with a correlation coefficient (R^2^) of 0.99. Reference gene stability was assessed using the BestKeeper algorithm [95] based on Ct values. RP15 (RNA polymerase I, II, and III, 15 kDa subunit) was selected as the reference gene due to its high expression stability (SD < 0.5). Expression levels were normalized to RP15 and calculated using the Pfaffl method [96]; the results are presented as relative expression values compared with the control.

Oxidative stress markers. The level of H_2_O_2_ was measured by a colorimetric method using an H_2_O_2_ Assay Kit (Elabscience, Wuhan, China). Detection principle: hydrogen peroxide can react with ammonium molybdate to form a colored compound. The optical density value was measured using a spectrophotometer at a wavelength of 405 nm. The content of H_2_O_2_ was calculated in mmol/g DW of leaves.

Intensity of lipid peroxidation in the leaves was determined as the content of MDA (TBARS) as described by Heath and Packer [97], with minor modifications. A leaf sample (300 mg) was homogenized in 5 mL of 0.35 M NaCl in 0.1 M Tris-HCl buffer, pH 7.6. The homogenate (3 mL) was mixed with 2 mL of 0.5% thiobarbituric acid in 20% trichloroacetic acid and then heated at 100 °C for 30 min, cooled, and filtered. The extraction medium containing the reagent was used as a control. Measurements were corrected for nonspecific absorbance by subtracting the values obtained at 532 nm and 600 nm. The content of MDA (TBARS) was calculated in μmol/g DW of leaves.

Enzymatic antioxidant activities. Leaf samples (500 mg) were homogenized in isolation medium (50 mmol Tris-HCl, pH 7.6; 3 mmol EDTA, 250 mmol sucrose, 3.6 mmol cysteine, 5 mmol ascorbic acid, 3 mmol MgCl_2_, 2 mmol DTT, 2 mmol PMSF), and centrifuged for 20 min at 16,000× *g*.

Superoxide dismutase (SOD, EC 1.15.1.1) activity was determined using a method based on the generation of superoxide radicals in the course of riboflavin photooxidation that is enhanced by an indicator trap (NBT, nitroblue tetrazolium) [98]. The reaction mixture contained 1.5% L-methionine, 0.14% NBT and 1% Triton X100 in a ratio of 3:1:0.75. Optical density measurements were performed at 560 nm. The inhibition of formazan production by 50% was recognized as a unit of SOD activity, which was converted to 1 mg of protein.

Catalase activity (CAT, EC 1.11.1.6) was measured by the rate of the H_2_O_2_ decomposition reaction [99]. The final reaction comprised 2.8 mL Tris-HCl buffer (10 mM, pH 7.6), 0.3 mL of enzyme extract and 0.3 mL of H_2_O_2_ (3 mM) for 3 min. The observed depletion of H_2_O_2_ in accordance with a drop in absorbance at 240 nm was used to measure CAT activity in the supernatant. CAT activity was expressed as μmol H_2_O_2_/mg protein min.

Guaiacol peroxidase activity (POX, EC 1.11.1.7) was determined according to Kumar and Knowles [99] with modifications. The method is based on oxidation of guaiacol to tetraguaiacol. The reaction mixture contained 0.3 mL of 0.15 mM guaiacol, 0.9 mL of Tris-HCl buffer (10 mM, pH 7.6) and 0.3 mL of the supernatant. Before optical density measurement, 0.3 mL of 0.1 mM H_2_O_2_ was added to the cuvette. The optical density was measured at 470 nm. The reaction was carried out at 25 °C for 1 min at intervals of 10 s. POX activity was expressed as μmol guaiacol/mg protein min.

The activity of glutathione reductase (GR, EC 1.6.4.2) was measured by the degree of NADPH oxidation according to the method of Foyer and Halliwell [100] with modifications. The reaction medium (3 mL) contained 8 µmol MgCl_2_·6H_2_O, 1.3 µmol GSSG and 0.7 µmol NADPH-Na_4_, which was prepared in 10 mM Tris-HCl buffer (pH 7.6) just before the reaction. The reaction was started by adding 0.2 mL of a supernatant containing glutathione reductase. The kinetics were recorded at 340 nm every 10 s for 2 min at a temperature of 30 °C. Corrections were made for any NADPH oxidation in the absence of GSSG. Enzyme activity was expressed in µmol NADPH(H+)/mg of protein per minute.

Non-enzymatic antioxidants and osmolytes. Proline content was determined using ninhydrin reagent and measured at 520 nm using a spectrophotometer [101]. An amount of 100 mg of leaves was ground in liquid nitrogen, added with 4 mL of distilled H_2_O, brought to a boil three times, and filtered. The resulting extract was used for analysis. Test tubes with 1 mL of extract, 1 mL of glacial acetic acid, and 1 mL of ninhydrin reagent (1.25 g ninhydrin, 20 mL of 6 M H_3_PO_4_, 30 mL of glacial acetic acid) were incubated for 1 h in a water bath. Proline content was expressed as mmol/g DW.

Quantitative determination of ascorbic acid (Asc) content in tissues was performed using potassium hexacyanoferrate [102]. A weighed sample of leaves (200 mg) was ground in liquid nitrogen, and 1.7 mL of citrate buffer (0.1 M, pH 3.69) containing 1.5 mmol Na-EDTA was added. The mixture was centrifuged for 5 min at 16,000× *g* and was used for further measurements. The reaction mixture contained: 500 µL of extract, 25 µL of 1% K_3_[Fe(CN)_6_], 25 µL of 2% NaF, 1.9 mL of deionized H_2_O, and 50 µL of 2% FeCl_3_. The resulting solution was incubated for 5–7 min, shaking periodically, after which the optical density was measured relative to the control (reaction mixture with buffer) at a wavelength of 680 nm. The concentration of ascorbic acid was determined using a calibration curve and expressed as mg/g DW.

Biological and statistical repetitions. For the concentration test, 7 concentrations of fullerenol were used, including the control (7 Petri dishes of 100 seeds each). The germination energy and seed germination were evaluated in the concentration test.

The concentration test was repeated two times. As a result, a fullerenol concentration of 0.1 mg/L was selected, which was used in further experiments.

Subsequently, 400 wheat seeds were used for each experiment (200 control seeds, 200 nanopriming seeds). Some of these plants, at the age of 10 days, were cooled at 4 °C for 5 days (approximately 100 control seedlings, 100 nanopriming seedlings). Growth and photosynthesis were measured on 30 plants. For electron microscopy, 5 biological replicates were used. In each of them, 10 cells were analyzed (a total of 50 cells per 1 experimental variant). All other physiological measurements were performed in 3 biological and 3 statistical repeats. These experiments were repeated three times.

*Statistical analyses.* The statistical significance of differences between means was assessed using ANOVA (Tukey’s test) built into the Microcal Origin 7.0 SRO (OriginLab Corporation, Northampton, MA, USA) graphical mathematical package. Data were expressed as means ± standard error (SE). In all cases, the confidence coefficient was set at 0.05. Correlation analysis, principal component analysis (PCA), and hierarchical cluster analysis were performed using the Anaconda 2025.12 distribution (Python 3.13.9), using the Pandas (2.3.3.), NumPy (2.3.5.), SciPy (1.16.3), Matplotlib (3.10.6), and Seaborn (0.13.2 ) libraries.

## 5. Conclusions

Considering the potential use of carbon-based nanomaterials in crop production, this study assessed the potential of PHF [C_60_(OH)_24_] as a metabolic modulator and adaptogen in wheat seedlings in optimal and low, non-damaging temperatures. In this research, the effect of pre-sowing seed treatment with PHF solutions of various concentrations on seed quality was studied. A concentration of 0.1 mg/L was selected for further studies. This concentration had the maximum effect on germination energy and percentage. Seedlings grown from PHF-primed wheat seeds differed from the control in accelerated growth of axial organs, increased biomass, larger chloroplasts and mesophyll cells, more intensive photosynthesis, increased content of chlorophyll *a*, soluble proteins and ascorbic acid, as well as reduced levels of MDA and H_2_O_2_ and reduced SOD activity against a background of higher levels of transcripts of cold-regulated genes. Under LT conditions, PHF priming stimulated photosynthesis by increasing the area of chloroplasts, the content of chlorophyll *b*, the proportion of chlorophyll in LHC, and *RBCS* expression. The pro-/antioxidant balance was maintained at or below the control level. This was accompanied by an increase in the content of proline and fructose, and an increase in the expression of COR genes. All these changes are adaptive, aimed at expanding the adaptive potential of plants. Along with data on an increase in growth processes under the influence of PHF, these results allow PHF to be considered an active ROS neutralizer and adaptogen.

## Figures and Tables

**Table 1:** Effect of seed priming with PHF (0.1 mg/L) on the structure of mesophyll cells in wheat seedlings in control and low-temperature conditions.

Parameter	23 °C		LT	
	Control	Nanopriming	Control	Nanopriming
S cell, µm^2^	245.5 ± 17.2 ^b^	300.0 ± 21.0 ^a^	296.9 ± 21.1 ^a^	282.4 ± 20.0 ^a^
S vac, µm^2^	148.2 ± 14.1 ^a^	161.4 ± 16.0 ^a^	145.9 ± 9.0 ^a^	146.2 ± 9.5 ^a^
S cyt, µm^2^	98.3 ± 9.8 ^b^	138.6 ± 12.8 ^a^	151.0 ± 14.2 ^a^	136.2 ± 12.3 ^a^
S vac, % from S cell	60 ± 2 ^a^	53 ± 2 ^b^	51 ± 2 ^b^	56 ± 2 ^ab^
S cyt, % from S cell	40 ± 2 ^b^	47 ± 2 ^a^	49 ± 2 ^a^	44 ± 2 ^ab^
N chp, pcs	9 ± 1 ^a^	10 ± 1 ^a^	9 ± 1 ^a^	9 ± 1 ^a^
S chp, µm^2^	7.8 ± 0.2 ^b^	8.4 ± 0.2 ^a^	8.8 ± 0.2 ^a^	9.0 ± 0.2 ^a^
S chp from S cyt, %	80 ± 3 ^a^	65 ± 3 ^b^	65 ± 3 ^b^	78 ± 3 ^a^

LT—exposure at 4 °C for 5 days. A total of 50 cells from 5 independent biological replicates were analyzed in each variant of treatment. Mean values ± SE are shown. In each row, values that differ significantly at *p* < 0.05 (Tukey’s test) are indicated by different letters. S cell—area of cell, S vac—area of vacuole, S cyt—area of cytoplasm, N chp—quantity of chloroplast, S chp—area of chloroplast.

**Table 2:** Effects of seed priming with PHF (0.1 mg/L) on PSA parameters and leaf dry matter content in wheat seedlings at 23 °C and after exposure to low (non-damaging) temperature.

Parameter	23 °C		LT	
	Control	Nanopriming	Control	Nanopriming
Net photosynthesis, mg CO_2_/(g DW h)	7.2 ± 0.9 ^b^	11.9 ± 1.1 ^a^	3.4 ± 0.4 ^d^	5.2 ± 0.6 ^c^
Chl *a*, mg/g DW	7.6 ± 0.1 ^c^	8.5 ± 0.1 ^b^	9.3 ± 0.1 ^a^	8.9 ± 0.1 ^a^
Chl *b*, mg/g DW	2.8 ± 0.1 ^c^	2.4 ± 0.1 ^c^	3.6 ± 0.1 ^b^	4.4 ± 0.1 ^a^
Car, mg/g DW	1.7 ± 0.1 ^a^	1.8 ± 0.1 ^a^	1.9 ± 0.1 ^a^	1.7 ± 0.1 ^a^
Chl *a*/*b*	2.7 ± 0.1 ^b^	3.5 ± 0.1 ^a^	2.6 ± 0.1 ^b^	2.1 ± 0.1 ^c^
Chl proportion in LHC, % DW content in 1 g of leaves, %	60 ± 2 ^b^ 14.4 ± 0.6 ^b^	49 ± 2 ^c^ 14.8 ± 0.8 ^b^	62 ± 2 ^b^ 16.8 ± 1.0 ^a^	73 ± 2 ^a^ 16.3 ± 1.1 ^a^

LT—exposure at 4 °C for 5 days, Chl—content of chlorophyll, Car—content of carotenoid, LHC—fraction of chlorophyll bound in the light-harvesting complex, DW—dry weight of shoot. In each row, values that differ significantly at *p* < 0.05 (Tukey’s test) are indicated by different letters.

**Table 3:** Primers for quantitative real-time PCR.

Gene	Primer	Sequences 5′→3′
*RP15*	Forward	TCATTGTGGAGGACTCGTGG
	Reverse	GCAGACATAGCCCACACAT
*WCOR726*	Forward	ACTGGAATGACCGGCTCG
	Reverse	TGTCCCGACTTCCCGTAGTT
*WCOR15*	Forward	CCACCCATCCATCAGCAGTT
	Reverse	CTTGGAGCGTTCTGCAGGC
*RBCS*	Forward	GGATTCGACAACATGCGCCAGG
	Reverse	ATATGGCCTGTCGTGAGTGAGC
*rbcL*	Forward	ACCATTTATGCGCTGGAGAGACC
	Reverse	CAAGTAATGCCCCTTGATTTCACC
*psbB*	Forward	GGTTTGCCTTGGTATCGTGT
	Reverse	TCCACATTGGATCCAGAACA
*psbD*	Forward	CGCTTTAGGGGGTGTGTTTA
	Reverse	GCCCCCATAGTAGCAACAAA
*psbH*	Forward	TGGCTACACAAACCGTTGAA
	Reverse	CCGTCCAGTAAAACGGAAGA

## Data Availability

The original contributions presented in this study are included in the article. Further inquiries can be directed to the corresponding author.

## Abbreviations

The following abbreviations are used in this manuscript:

GE	germination energy
GP	germination percentage
GR	glutathione reductase
MDA	malondialdehyde
PCA	principal component analysis
PHF C_60_(OH)_24_	derivative of fullerene C60 carrying twenty-four hydroxyl groups
POX	guaiacol peroxidase
PSII	photosystem II
ROS	reactive oxygen species
SOD	superoxide dismutase
COR gene	cold-regulated genes
CAT	catalase
Asc	ascorbic acid
APX	ascorbate peroxidase
TBARS	thiobarbituric acid reactive substances
RuBisCo	ribulose-1,5-bisphosphate carboxylase/oxygenase
